# Higher HEI-2015 score is associated with reduced risk of Parkinson’s disease: a nationwide population-based study

**DOI:** 10.3389/fnut.2025.1541271

**Published:** 2025-05-30

**Authors:** Wenting Hu, Hai Liu, Ying Zhang, Huanxian Liu

**Affiliations:** ^1^Department of Neurology, Chengdu Integrated TCM and Western Medicine Hospital, Chengdu First People’s Hospital, Chengdu, Sichuan, China; ^2^Department of Neurology, Xuanhan County People’s Hospital, Dazhou, Sichuan, China; ^3^Department of Anus and Intestine Surgery, Chengdu Integrated TCM and Western Medicine Hospital, Chengdu First People’s Hospital, Chengdu, Sichuan, China; ^4^Department of Neurology, The First Medical Center, Chinese PLA General Hospital, Beijing, China; ^5^International Headache Centre, Chinese PLA General Hospital, Beijing, China

**Keywords:** healthy eating index, diet, Parkinson’s disease, NHANES, cross-sectional study

## Abstract

**Background:**

Recent studies have highlighted the significant role of diet in the development of Parkinson’s disease (PD). However, research on the association between diet quality and PD in the general adult population of the United States remains limited. This study aims to assess the relationship between diet quality, measured by the Healthy Eating Index 2015 (HEI-2015) score, and the risk of PD.

**Methods:**

Data for this cross-sectional analysis were obtained from the National Health and Nutrition Examination Survey (NHANES) from 2003 to 2018, which includes a nationally representative sample of US adults. Diet quality was measured using the HEI-2015 score, and weighted multivariable logistic regressions and restricted cubic splines (RCS) were applied to examine the correlation between HEI-2015 and PD. Threshold effects were computed using a two-segment linear regression model. Subgroup and sensitivity analyses, including multiple imputations, unweighted logistic regression, and exclusion of participants with HEI-2015 scores beyond 3 standard deviations (mean ± 3SD), were performed to assess the robustness of the findings.

**Results:**

A total of 29,581 US adults were included in the analysis, with 286 participants diagnosed with PD. In the fully adjusted multivariable model, each 10-point increase in the HEI-2015 score was associated with a 17% reduction in the likelihood of PD (odds ratio (OR):0.858,95% confidence interval (CI):0.742–0.992, *p* = 0.039). Additionally, individuals with higher HEI-2015 scores had a 62% lower probability of developing PD compared to those with lower scores (OR:0.518, 95%CI:0.297–0.906, *p* = 0.021). RCS analysis revealed a nonlinear relationship between HEI-2015 scores and PD (*p* = 0.022). In the two-segment regression models, participants with HEI-2015 scores ≥ 55.500 had an adjusted OR of 0.957 for developing PD (95% CI: 0.916–0.999, *p* = 0.045). In contrast, no association was observed between HEI-2015 scores and PD in participants with scores < 55.500. Subgroup analyses indicated the association was modified by race and hyperlipidemia (*P* for interaction = 0.039 and 0.024, respectively). Sensitivity analyses further confirmed the robustness of this association.

**Conclusion:**

HEI-2015 is negatively associated with the prevalence of PD. This suggests that modifiable lifestyle factors, particularly diet quality, may play an important role in reducing the risk of PD.

## 1 Introduction

Parkinson’s disease (PD) is one of the neurodegenerative diseases with the fastest-growing prevalence worldwide (1). The number of people with PD is predicted to grow significantly within the next few decades due to the persistent global aging trend (2). PD may lead to severe disability and impair quality of life while also imposing a significant economic burden and placing considerable pressure on healthcare systems (1, 3, 4). Although extensive research into the pathophysiology and treatment of PD, no definitive cure has been identified. Early diagnosis and preventive strategies are crucial. Identifying modifiable risk factors that influence the onset and progression of PD is essential for effective intervention (5–7). Recent studies have suggested that various dietary factors, including magnesium, *β*-carotene, niacin, selenium, added sugars, iron intake, western dietary patterns, and the Mediterranean diet (MeDi), are associated with PD (6, 8–15). These findings suggest that diet may serve as an essential component in PD prevention.

The Healthy Eating Index 2015 (HEI-2015) is a well-known instrument to evaluate the overall quality of diets because of the importance of diet in health outcomes. It was developed on the 2015–2020 Dietary Guidelines for Americans (DGA), and it has also been recommended for use during the 2020–2025 period (16, 17). It evaluates a broad range of dietary components, providing a comprehensive measure of dietary patterns. Specifically, HEI-2015 assesses 13 distinct dietary components, offering a more nuanced understanding of complex eating habits compared to other dietary assessment tools. It has been demonstrated to provide a more profound understanding of the connection between diet and health (18–22). Notably, studies have linked HEI-2015 scores to several neurological disorders, including stroke, depression, and cognitive decline function (23–25). However, much of the existing research has focused on specific aspects of diet, such as individual nutrients, food groups, or isolated dietary patterns on PD. In contrast, the broader 92 relationship between overall diet quality, as measured by HEI-2015, and the risk of PD remains insufficiently explored. This gap highlights the need to investigate how general diet quality may influence the onset and progression of PD.

This study employs a cross-sectional analysis, utilizing data collected from the National Health and Nutrition Examination Survey (NHANES) from 2003 to 2018. Our objective was to apply a large, nationally representative sample from the NHANES database to investigate the relationship between HEI-2015 scores and PD. Our hypothesis was that higher HEI-2015 scores would be related to a decreased risk of PD. Identifying dietary patterns associated with PD could inform public health strategies and clinical guidelines, offering valuable insights for both the prevention and management of PD.

## 2 Materials and methods

### 2.1 Data source

The NHANES is a comprehensive, ongoing study that evaluates the nutritional status and general health of the citizens of the United States who are not institutionalized. This nationally representative survey utilizes a stratified, multistage design to ensure the data accurately reflects the broader U.S. demographic. NHANES collects detailed information across various domains, including demographics, socioeconomic status, dietary intake, and medical conditions. Data collection primarily occurs through home interviews, which are supplemented by laboratory tests, including blood analysis (26).

The NHANES documentation contains comprehensive documentation of the study’s methodology and analytical procedures that are accessible to the public.^1^ The National Center for Health Statistics (NCHS) ethical review board examined and consented to the NHANES procedures. After enrolling, participants in the survey furnish their written informed permission. There was no need for extra ethical approval or participant permission because this study used deidentified data that was publicly accessible.

### 2.2 Study design and population

**Figure 1:**
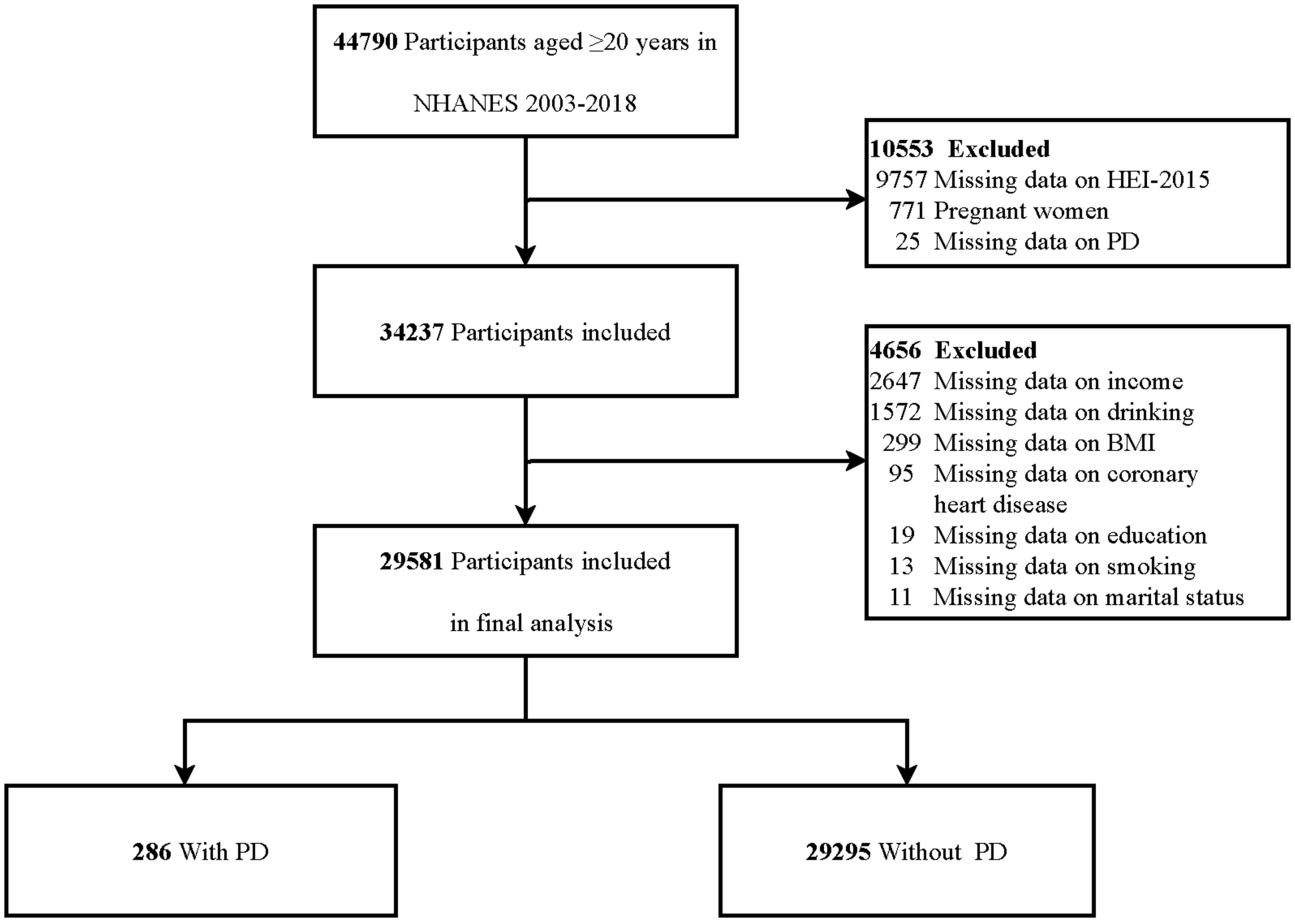
Flowchart of participants selection. HEI-2015, Healthy Eating Index 2015; BMI, body mass index; PD, Parkinson’s disease.

This study employs a cross-sectional design, utilizing data from eight NHANES survey cycles conducted between 2003 and 2018. The analysis included individuals aged 20 years and older who had completed the survey interview with available dietary assessments and PD-related data. Several exclusion criteria were applied to ensure the validity of the dataset. Participants were excluded if they were pregnant, had missing HEI-2015 data, had missing data on PD, or lacked data on relevant covariates. Figure 1 presents the specific inclusion and exclusion criteria in detail.

### 2.3 Assessment of HEI-2015

The primary exposure in this study was HEI-2015, a commonly used instrument to assess the quality of diets. Detailed dietary intake data were collected from NHANES participants through two 24-h dietary recalls. The first dietary recall was conducted during an in-person interview at the Mobile Examination Center (MEC), and the second recall took place by telephone 3 to 10 days later. These recalls were intentionally scheduled on different days of the week, including both weekdays and weekends, to capture intra-individual variation in dietary intake. NHANES adjusts for potential day-of-week biases using specialized weights: WTDRD1 for single-day data and WTDR2D for two-day data, ensuring proportional representation of weekday and weekend recall combinations.^2^ These two sets of dietary information were combined and used to assess overall diet quality and calculate various dietary quality indicators. The HEI-2015 score, which ranges from 0 to 100, comprises 13 components. The components are divided into two categories: nine adequacy components, which include total fruits, whole fruits, vegetables, greens and beans, total protein foods, seafood and plant proteins, whole grains, dairy, and fatty acids (each scored between 0–5 and 0–10 points), and four moderation components, which include sodium, refined grains, added sugars, and saturated fats (each scored from 0 to 10 points) (17). A higher HEI-2015 score indicates a higher-quality diet and greater adherence to dietary recommendations. Two dietary recalls were conducted on participants in order to have an extensive overview of their daily food intake. The mean of these two recalls (DR1TOT and DR2TOT) was used for analysis (27, 28).

Given the wide range of possible HEI-2015 scores, which span from 0 to 100, the effect of each incremental change in the HEI-2015 score on PD prevalence is relatively small. Therefore, we initially examined the association between a 10-point increase in the HEI-2015 score and PD prevalence. Furthermore, to explore the relationship between dietary quality and PD risk across different dietary quality levels, the HEI-2015 scores were divided into quartiles, following the approach used in previous studies (18, 19, 21, 22, 29). These quartile boundaries were as follows: Q1 (<44.264), Q2 (44.265–53.207), Q3 (53.208–62.857), and Q4 (≥62.858).

Detailed criteria for scoring the HEI-2015 are provided in Supplementary Table S1.

### 2.4 Assessment of PD

PD was the outcome variable in this study. The criteria for defining PD were consistent with established definitions in the literature (9, 30, 31). To identify participants with PD, data from the Prescription Medications records, specifically prescriptions categorized as “ANTIPARKINSON AGENTS,” were used. This classification was based on participants’ self-reported use of prescribed medications. Due to limitations in the NHANES dataset, only individuals actively treated for PD with antiparkinsonian medications were classified as having PD. Participants not using these medications were considered non-PD.

### 2.5 Covariates

In this study, various covariates were adjusted based on established research (9, 12, 31, 32). These included demographic factors such as age, sex, race/ethnicity (non-Hispanic White, non-Hispanic Black, Mexican American, other Hispanic, and other races) and marital status (married/living with a partner vs. living alone, including never married, separated, divorced, and widowed). Socioeconomic factors, including family income and education level, were also considered.

Age was categorized into two groups: <60 and ≥60 years. Family income was categorized based on the poverty income ratio (PIR) into three groups: low (PIR < 1.3), medium (PIR 1.3–3.5), and high (PIR ≥ 3.5). Education level was classified as less than high school, high school or equivalent, and above high school.

Lifestyle factors, such as smoking status and alcohol consumption, were incorporated. By existing literature, smoking status was defined as never smokers (having smoked fewer than 100 cigarettes in their lifetime), former smokers (having smoked more than 100 cigarettes but having since quit), or current smokers (having smoked more than 100 cigarettes and still smoking). Alcohol consumption was classified into five categories: never drinkers (individuals who have never consumed alcohol), former drinkers (those who have consumed alcohol at least once but did not drink in the past year), mild drinkers (≤1 drink per day for females or ≤2 drinks per day for males), moderate drinkers (≥2 drinks per day for females, ≥3 drinks per day for males, or engaging in binge drinking on ≥2 days per month), and heavy drinkers (≥3 drinks per day for females, ≥4 drinks per day for males, or binge drinking on ≥5 days per month).

Anthropometric and physical activity data were also included. Body mass index (BMI) was categorized as <25, 25–30, and ≥30 kg/m^2^. Physical activity (PA) was measured using MET-minutes per week, incorporating activity type, frequency, and duration. MET values for different activities were obtained from the NHANES database, and PA was calculated using the following formula: PA (MET-min/week) = MET × weekly frequency × duration (33, 34).

Additionally, certain self-reported medical conditions were considered, including coronary heart disease (diagnosed by a doctor), hyperlipidemia, and diabetes (all classified as yes/no). Hyperlipidemia was defined by any of the following: TG ≥ 150 mg/dL, total cholesterol ≥200 mg/dL, LDL-C ≥ 130 mg/dL, HDL-C < 40 mg/dL, or current use of hypolipidemic medications. Diabetes was diagnosed based on any of the following: self-reported doctor’s diagnosis, HbA1c > 6.5%, fasting glucose > 7.0 mmol/L, random glucose > 11.1 mmol/L, 2-h glucose tolerance test > 11.1 mmol/L, or use of insulin or diabetic medications.

### 2.6 Statistical analysis

In accordance with NHANES analysis guidelines (26, 35), this study accounted for the complex sampling design and sampling weights. We used dietary weights recommended by the Centers for Disease Control and Prevention (CDC).^3^ Specifically, the second-day dietary sample weight (WTDRD2) from NHANES 2003–2018 data was applied. The exact sampling weight used was 1/8 WTDRD2.

In the baseline characteristics table, continuous variables with a normal distribution were presented as means and standard deviations (SD). Values for non-normal continuous variables were presented as the median and interquartile range (IQR). Cases (n) and percentages (%) were used to display categorical variables. The chi-square test for categorical variables across groups and the one-way analysis of variance (ANOVA) for continuous variables with a normal distribution were the suitable tests used for statistical comparisons. We used the Kruskal-Wallis rank sum test for continuous variables with non-normal distributions. Given the large sample size in this study, missing data were managed by excluding incomplete records from the analysis. To examine the correlation between HEI-2015 scores and PD, weighted multivariable logistic regression was employed. This method was used to calculate the odds ratios (ORs) and 95% confidence intervals (95% CIs) for the association between a 10-point increase in HEI-2015 scores, categorical variables (which were divided into quartiles), and PD risk while adjusting for potential confounders. The regression models were as follows: Model 1, unadjusted. Model 2, adjusted for age, sex, race, marital status, family income, and educational level. Model 3, adjusted for variables in Model 2, with additional adjustments for smoking status, alcohol consumption, PA, and BMI. Model 4, further adjusted for coronary heart disease, hyperlipidemia, and diabetes, in addition to the factors in Model 3. The same analytical methodology was applied to explore the associations between the 13 distinct elements of the HEI-2015 and PD risk.

Using a three-knot limited cubic spline, the weighted restricted cubic spline model was utilized to evaluate the dose–response association between HEI-2015 and PD. A two-piecewise logistic regression model was established to examine the connection. Confounders included in Model 4 were adjusted for in the analysis.

To assess whether the relationship between HEI-2015 scores and PD remains consistent across different populations, interaction and subgroup analyses were performed. These analyses were based on age groups (<60 vs. ≥60 years), sex (male vs. female), ace (non-Hispanic White vs. other races), marital status (married or living with partners vs. living alone), smoking status (never smokers vs. former or current smokers), BMI categories (<25 vs. ≥25 kg/m^2^), and presence of hyperlipidemia (no vs. yes). Logistic regression models and likelihood ratio tests were used to assess heterogeneity and interactions across these subgroups.

### 2.7 Sensitivity analyses

To enhance the robustness of our findings, multiple imputations were employed to address missing covariates data, thus reducing potential selection bias that could arise from excluding participants with incomplete information. This approach generated five complete datasets, and the results from these datasets were then aggregated. Additionally, a sensitivity analysis was conducted using unweighted logistic regression to further examine the relationship between HEI-2015 scores and PD. To guarantee the analysis’s validity, extreme values of HEI-2015 scores (those outside the range of mean ± 3SD) were excluded. The association between PD and HEI-2015 scores was then examined using the weighted multivariate logistic regression model previously indicated.

R version 4.2.2 and Free Statistics software version 2.0 (Beijing, China) were used for statistical analyses. All participants were included in a descriptive study, and a two-sided *p*-value of less than 0.05 was considered statistically significant. The data were analyzed from August to November 2024.

## 3 Results

### 3.1 Study population

In the NHANES study conducted from 2003 to 2018, which involved 44,790 individuals aged 20 years or older, 15,209 participants were excluded. Specifically, 771 individuals were excluded due to pregnancy, 9,757 lacked HEI-2015 data, 25 had missing information on PD, and 4,656 had unavailable covariate data. Consequently, the final analysis included 29,581 participants (Figure 1).

### 3.2 Baseline characteristics of the participants

The weighted distribution and baseline characteristics of participants, categorized by HEI-2015 quartiles, are presented in Table 1. The final analysis included a total of 29,581 individuals, which represents approximately 193.88 million adults in the United States aged 20 years or older. The research population’s weighted mean age was 47.57 years (standard deviation (SD) = 16.87), with 48.76% of participants identifying as male and 51.24% as female. Participants in the higher quartiles of HEI-2015 scores tended to be older, more likely to be female, non-Hispanic White, and married or living with a partner. They also reported mild alcohol consumption, had a lower BMI, and had higher family incomes. Additionally, higher education levels and higher metabolic equivalent (MET) values for PA were also associated with participants in the higher quartiles. Participants with higher HEI-2015 scores had a prevalence rate of 0.54%.

**Table 1:** Characteristics of participants by categories of HEI-2015: NHANES 2003–2018, weighted.

Characteristics	Total (weighted)	Quartiles (Q) of HEI-2015				*p-*value
		Q1 (<44.086)	Q2 (44.087–53.055)	Q3 (53.056–62.714)	Q4 (≥62.714)	
NO. (10,000)	19388.51	5163.89	4844.26	4694.48	4685.89	
Age (years), mean (SD)	47.57 (16.87)	43.08 (16.10)	46.54 (16.54)	48.96 (16.93)	52.19 (16.60)	<0.0001
Sex, *n* (%)						
Male	9453.39 (48.76)	2810.62 (54.43)	2482.44 (51.25)	2180.26 (46.44)	1980.06 (42.26)	<0.0001
Female	9935.12 (51.24)	2353.26 (45.57)	2361.82 (48.75)	2514.22 (53.56)	2705.82 (57.74)	
Race, *n* (%)						
Non-Hispanic White	13551.75 (69.90)	3606.40 (69.84)	3324.15 (68.62)	3255.49 (69.35)	3365.72 (71.83)	<0.0001
Non-Hispanic Black	2064.75 (10.65)	670.14 (12.98)	566.79 (11.70)	480.81 (10.24)	347.00 (7.41)	
Mexican American	1533.12 (7.91)	413.00 (8.00)	405.58 (8.37)	395.79 (8.43)	318.75 (6.80)	
Other Hispanic	915.27 (4.72)	203.26 (3.94)	216.23 (4.46)	238.07 (5.07)	257.70 (5.50)	
Other	1323.62 (6.83)	271.08 (5.25)	331.51 (6.84)	324.32 (6.91)	396.72 (8.47)	
Marital status, *n* (%)						
Married or Living with a partner	12303.16 (63.46)	3072.81 (59.51)	3056.85 (63.10)	3025.44 (64.45)	3148.06 (67.18)	<0.0001
Living alone	7085.34 (36.54)	2091.07 (40.49)	1787.40 (36.90)	1669.04 (35.55)	1537.82 (32.82)	
Family income, *n* (%)						
≤1.30	3998.80 (20.62)	1344.11 (26.03)	1069.42 (22.08)	941.63 (20.06)	643.64 (13.74)	<0.0001
1.31–3.50	6868.81 (35.43)	1989.39 (38.53)	1767.45 (36.49)	1607.34 (34.24)	1504.63 (32.11)	
>3.50	8520.89 (43.95)	1830.38 (35.45)	2007.39 (41.44)	2145.50 (45.70)	2537.62 (54.15)	
Education level, *n* (%)						
Less than high school	2783.09 (14.35)	926.16 (17.94)	725.29 (14.97)	636.93 (13.57)	494.71 (10.56)	<0.0001
High school or equivalent	4574.63 (23.59)	1552.75 (30.07)	1258.01 (25.97)	1005.70 (21.42)	758.17 (16.18)	
Above high school	12030.78 (62.05)	2684.98 (52.00)	2860.96 (59.06)	3051.85 (65.01)	3433.00 (73.26)	
Smoking status, *n* (%)						
Never	10577.50 (54.56)	2499.34 (48.40)	2538.78 (52.41)	2656.09 (56.58)	2883.29 (61.53)	<0.0001
Former	4912.28 (25.34)	1031.06 (19.97)	1176.35 (24.28)	1309.67 (27.90)	1395.21 (29.77)	
Now	3898.73 (20.11)	1633.49 (31.63)	1129.13 (23.31)	728.71 (15.52)	407.39 (8.69)	
Drinking status, *n* (%)						
Never	2036.45 (10.50)	478.45 (9.27)	486.87 (10.05)	502.22 (10.70)	568.9 (12.14)	<0.0001
Former	3048.55 (15.72)	943.62 (18.27)	764.07 (15.77)	694.55 (14.80)	646.30 (13.79)	
Mild	6978.98 (36.00)	1574.63 (30.49)	1633.36 (33.72)	1747.14 (37.22)	2023.86 (43.19)	
Moderate	3360.57 (17.33)	876.83 (16.98)	829.98 (17.13)	841.10 (17.92)	812.67 (17.34)	
Heavy	3963.96 (20.44)	1290.36 (24.99)	1129.98 (23.33)	909.46 (19.37)	634.16 (13.53)	
BMI (kg/m^2^),mean (SD)	29.01 (6.84)	29.85 (7.43)	29.30 (6.89)	28.96 (6.68)	27.82 (6.05)	<0.0001
Coronary heart disease, *n* (%)						
No	18675.61 (96.32)	5005.87 (96.94)	4692.73 (96.87)	4514.61 (96.17)	4462.40 (95.23)	0.0003
Yes	712.89 (3.68)	158.02 (3.06)	151.53 (3.13)	179.86 (3.83)	223.48 (4.77)	
Hyperlipidemia, *n* (%)						
No	5923.40 (30.55)	1568.32 (30.37)	1524.08 (31.46)	1403.02 (29.89)	1427.99 (30.47)	0.5799
Yes	13465.11 (69.45)	3595.57 (69.63)	3320.18 (68.54)	3291.46 (70.11)	3257.90 (69.53)	
Diabetes, *n* (%)						
No	16784.83 (86.57)	4554.91 (88.21)	4195.99 (86.62)	4029.57 (85.84)	4004.36 (85.46)	0.0021
Yes	2603.68 (13.43)	608.98 (11.79)	648.27 (13.38)	664.91 (14.16)	681.52 (14.54)	
Parkinson, n (%)						
No	19214.47 (99.10)	5114.42 (99.04)	4794.76 (98.98)	4644.61 (98.94)	4660.68 (99.46)	0.1342
Yes	174.04 (0.90)	49.46 (0.96)	49.50 (1.02)	49.878 (1.06)	25.21 (0.54)	
PA (MET-min/wk), median (IQR)	960.00 (120.00,3360.00)	819.00 (60.00, 3855.69)	759.83 (63.00, 3360.00)	900.00 (91.00, 2880.00)	1200.00 (260.40,3360.00)	<0.0001

Mean ± SD for continuous variables, *p*-value was by weighted linear regression. % for categorical variables, the *P*-value was by survey-weighted Chi-square test. Q1 represents the unhealthiest diet quality, and Q4 represents the healthiest diet quality. *p* < 0.05 presents a significant difference. HEI-2015, Healthy Eating Index 2015; BMI, body mass index; PA, physical activity.

Participants with PD were older than those without PD, with a mean age of 61.21 years (SD = 16.01) compared to non-PD participants. They also had a higher BMI, with a mean BMI of 30.56 (SD = 7.20) compared to 29.27 (SD = 6.94) in non-PD participants. Additionally, participants with PD had lower MET values than those without PD (Supplementary Table S2).

### 3.3 Association between HEI-2015 and PD

The correlation between HEI-2015 scores and PD risk was evaluated using weighted logistic regression models, with results presented in Table 2.

**Table 2:** Association of HEI-2015 with PD, weighted.

Variables	Model 1		Model 2		Model 3		Model 4	
	OR (95%CI)	*p*-value	OR (95%CI)	*p*-value	OR (95%CI)	*p*-value	OR (95%CI)	*p*-value
HEI-2015 Per 10-points increase	0.881 (0.779–0.997)	0.045	0.816 (0.713–0.934)	0.003	0.858 (0.743–0.992)	0.038	0.858 (0.742–0.992)	0.039
Quartile (Q) of HEI-2015								
Q1 (<44.086)	1(Ref)				1(Ref)		1(Ref)	
Q2 (44.087–53.053)	1.067 (0.663–1.771)	0.787	0.972 (0.602–1.569)	0.906	1.034 (0.640–1.672)	0.889	1.035 (0.637–1.682)	0.888
Q3 (53.054–62.714)	1.110 (0.640–1.926)	0.708	0.916 (0.518–1.618)	0.760	1.038 (0.565–1.908)	0.904	1.036 (0.565–1.898)	0.907
Q4 (≥62.715)	0.559 (0.332–0.943)	0.030	0.436 (0.255–0.744)	0.003	0.520 (0.298–0.908)	0.022	0.518 (0.297–0.906)	0.021
*P* for trend		0.072		0.007		0.067		0.064

Model 1, adjusted for none. Model 2, adjusted for age, sex, race, marital status, family income, and educational level. Model 3, adjusted for age, sex, race, marital status, family income, educational level, smoking status, drinking status, physical activity, and BMI. Model 4, adjusted for age, sex, race, marital status, family income, educational level, smoking status, drinking status, physical activity, BMI, coronary heart disease, hyperlipidemia, and diabetes. BMI, body mass index; OR, odds ratio; CI, confidence interval; Ref, reference; HEI-2015, Healthy Eating Index-2015; PD, Parkinson’s disease.

A 10-point increase in HEI-2015 score was negatively associated with PD risk across all models. In Model 1 (unadjusted), OR was 0.881 (95% CI: 0.779–0.997, *p* = 0.045). In Model 2 (minimally adjusted), the OR was 0.816 (95% CI: 0.713–0.934, *p* = 0.003), and in Model 3, the OR remained 0.858 (95% CI: 0.743–0.992, *p* = 0.038). In the fully adjusted model (Model 4), the OR was 0.858 (95% CI: 0.742–0.992, *p* = 0.039).

Using quartiles of HEI-2015 scores, higher scores were associated with a lower PD prevalence in all models. In all models, individuals in the fourth quartile showed a lower prevalence of PD than those in the first: Model 1 (OR: 0.559, 95% CI: 0.332–0.943, *p* = 0.030), Model 2 (OR: 0.436, 95% CI: 0.255–0.744, *p* = 0.003), Model 3 (OR: 0.520, 95% CI: 0.298–0.908, *p* = 0.022), and Model 4 (OR: 0.518, 95% CI: 0.297–0.906, *p* = 0.021).

### 3.4 Association of components of HEI-2015 with PD

Logistic regression models were used to assess the relationship between individual components of the HEI-2015 and the risk of PD. In Model 4, several components showed a significant negative association with PD risk, including total vegetables (OR: 0.812, 95% CI: 0.706–0.933, *p* = 0.004), greens and beans (OR: 0.884, 95% CI: 0.803–0.973, *p* = 0.013), total protein foods (OR: 0.811, 95% CI: 0.697–0.942, *p* = 0.007), seafood and plant proteins (OR: 0.922, 95% CI: 0.859–0.990, *p* = 0.027), and added sugars (OR: 0.941, 95% CI: 0.893–0.991, *p* = 0.022). Conversely, sodium score was positively associated with PD risk (OR: 1.071, 95% CI: 1.006–1.114, *p* = 0.031). No significant correlations were identified with other HEI-2015 components (Supplementary Table S3).

### 3.5 Dose–response non-linear relationship analysis

**Figure 2:**
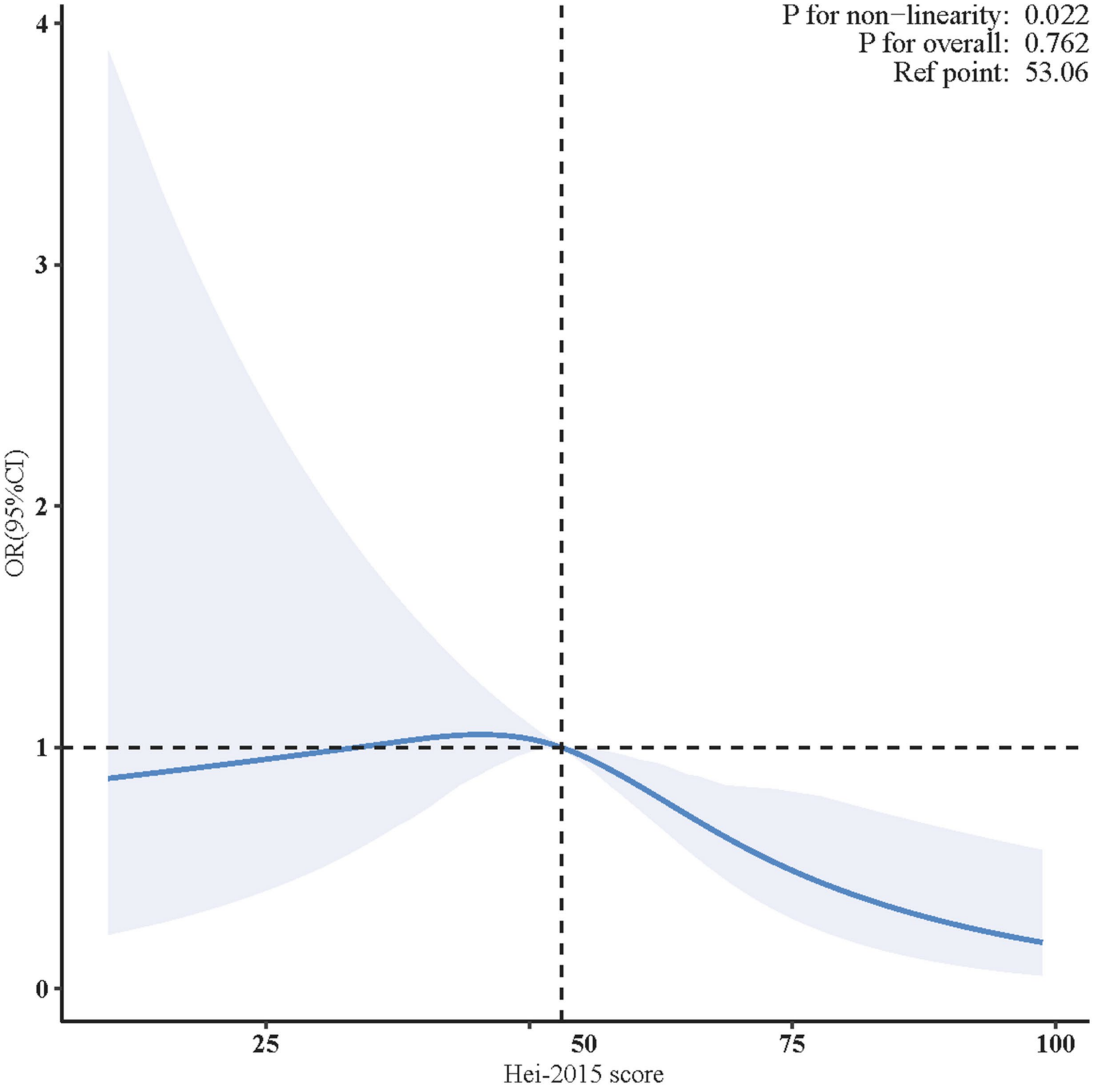
Non-linear restricted cubic spline (RCS) plot between HEI-2015 and PD. The blue line indicates the dose–response relationship between HEI-2015 and PD. The blue area represents the 95% confidence interval. Adjusted for age, sex, race, marital status, family income, educational level, smoking status, drinking status, physical activity, BMI, coronary heart disease, hyperlipidemia, and diabetes. BMI, body mass index; OR, odds ratio; CI, confidence interval; HEI-2015, Healthy Eating Index-2015; PD, Parkinson’s disease.

As illustrated in Figure 2, the non-linearity test’s *p* value was 0.022, indicating a non-linear correlation between HEI-2015 and PD. The analysis was adjusted for confounders, including age (as a continuous variable), sex, race, marital status, family income, educational level, smoking status, drinking status, physical activity, BMI, coronary heart disease, hyperlipidemia, and diabetes.

In the two-piecewise regression model, a statistically reverse association between HEI-2015 scores and PD risk was observed only in participants with HEI-2015 scores ≥ 55.500 (adjusted OR = 0.957, 95% CI: 0.916–0.999, *p* = 0.045). No significant relationship was found for those with HEI-2015 scores < 55.500 (Table 3).

**Table 3:** Threshold effect analysis of the relationship of HEI-2015 with PD.

HEI-2015	Adjusted model	
	OR (95%CI)	*p*-value
<55.500	0.985 (0.956–1.014)	0.308
≥55.500	0.957 (0.916–0.999)	0.045

HEI-2015, Healthy Eating Index-2015; OR, odds ratio; CI, confidence intervals; PD, Parkinson’s disease.

### 3.6 Subgroup analyses

**Figure 3:**
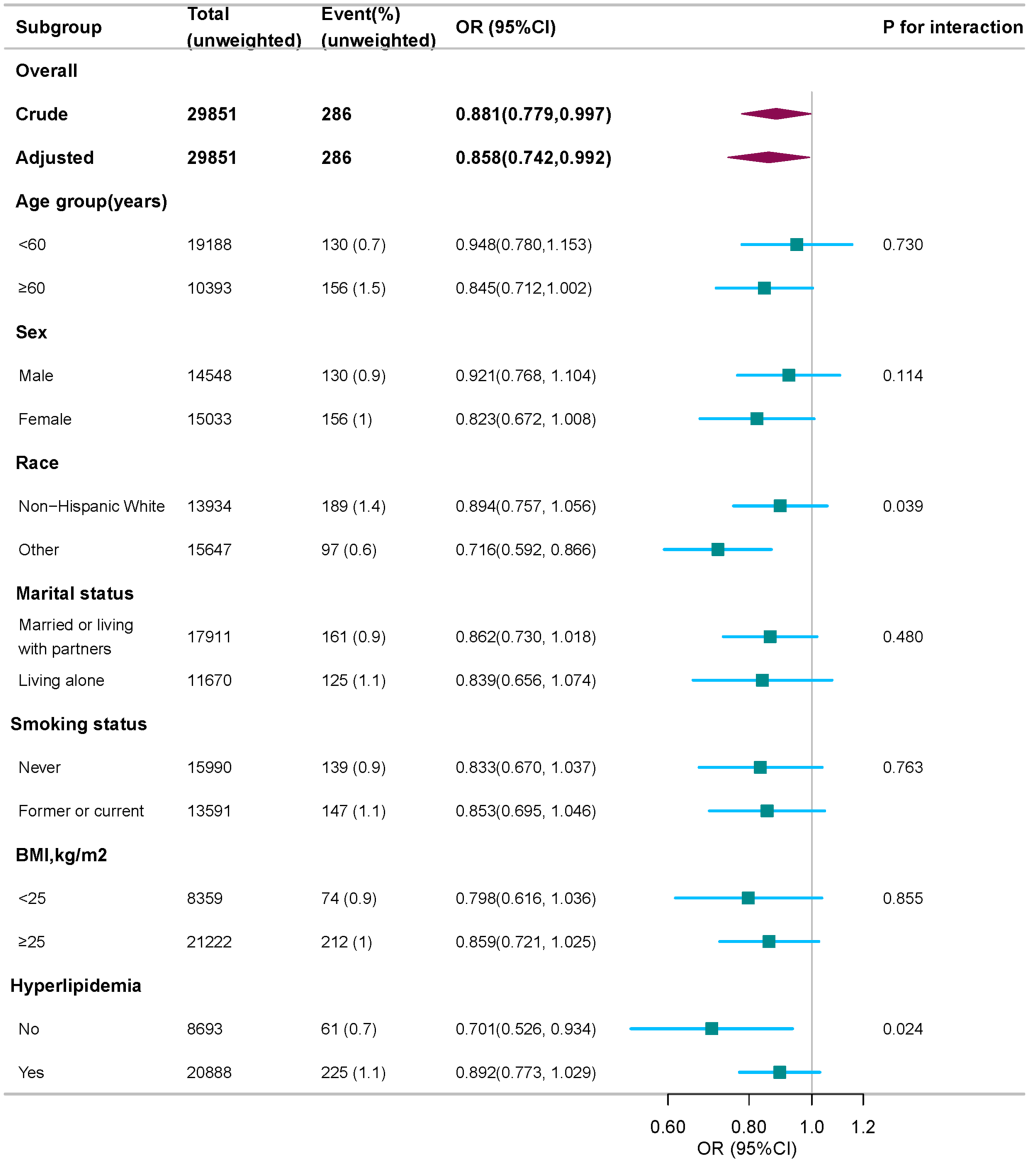
Subgroup analysis of the association of HEI-2015 with PD, weighted. Odds ratios (ORs) were calculated as per 10 scores increase in HEI-2015 score. Except for the stratification factor itself, the stratifications were adjusted for age, sex, race, marital status, family income, educational level, smoking status, drinking status, physical activity, BMI, coronary heart disease, hyperlipidemia, and diabetes. BMI, body mass index; OR, odds ratio; CI, confidence interval; HEI-2015, Healthy Eating Index-2015; PD, Parkinson’s disease.

In order to evaluate the consistency of the relationship between each 10-unit increase in HEI-2015 scores and PD risk across different populations, we performed subgroup analyses using the fully adjusted logistic regression model. Stratification by age, sex, marital status, BMI, and smoking status revealed no statistically significant interactions (all *P* for interaction > 0.05, Figure 3). However, race and hyperlipidemia were identified as effect modifiers. Results showed significant differences in non-Hispanic Whites and other races (OR = 0.894 and 0.716, respectively; *P* for interaction = 0.039).

Furthermore, a significant protective association was found between HEI-2015 scores and PD risk in individuals without hyperlipidemia (OR = 0.701, 95% CI: 0.526–0.934, *P* for interaction = 0.024). However, no significant association was observed in individuals with both PD and hyperlipidemia, suggesting that the protective effect of a high-quality diet may be weakened in this group.

### 3.7 Sensitivity analyses

Initially, we conducted multiple imputations for missing covariate data. We generated five imputed datasets and conducted multivariable logistic regression to verify the accuracy and reliability of our results. In model 4, for every 10-point rise in the HEI-2015 score, the likelihood of PD prevalence dropped by 15.1% (OR:0.849, 95%CI:0.777–0.928, *p* < 0.001). Stratification by HEI-2015 quartiles showed that participants in higher quartiles had a lower risk of PD. Specifically, the odds of having PD were considerably lower for Q3 (OR = 0.709, 95% CI: 0.52–0.966, *p* = 0.029) and Q4 (OR = 0.582, 95% CI: 0.418–0.811, *p* = 0.001) (Table 4).

**Table 4:** Association between HEI-2015 and PD after multiple imputations.

Variables	Unweighted participants/total participants, No.		Model 1		Model 2		Model 3		Model 4	
	Without PD	With PD	OR (95%CI)	*p*-value	OR (95%CI)	*p*-value	OR (95%CI)	*p*-value	OR (95%CI)	*p*-value
HEI-2015 Per 10-points increase	33,895/34,237	342/34,237	0.888 (0.819–0.964)	0.005	0.800 (0.730–0.876)	<0.001	0.818 (0.743–0.901)	<0.001	0.849 (0.777–0.928)	<0.001
Quartile (Q) of HEI-2015										
Q1 (<44.264)	8,461/8,559	98/342	1(Ref)		1(Ref)		1(Ref)		1(Ref)	
Q2 (44.265–53.207)	8,459/8,559	100/342	1.021 (0.771–1.351)	0.886	0.962 (0.717–1.291)	0.795	0.968 (0.713–1.313)	0.833	0.957 (0.72–1.273)	0.764
Q3 (53.207–62.857)	8,482/8,559	77/342	0.784 (0.581–1.058)	0.111	0.653 (0.473–0.902)	0.009	0.668 (0.476–0.936)	0.019	0.709 (0.52–0.966)	0.029
Q4 (≥62.858)	8,493/8,560	67/342	0.681 (0.498–0.931)	0.016	0.487 (0.344–0.689)	<0.001	0.521 (0.361–0.752)	<0.001	0.582 (0.418–0.811)	0.001

Model 1, adjusted for none. Model 2, adjusted for age, sex, race, marital status, family income, and educational level. Model 3, adjusted for age, sex, race, marital status, family income, educational level, smoking status, drinking status, physical activity, and BMI. Model 4, adjusted for age, sex, race, marital status, family income, educational level, smoking status, drinking status, physical activity, BMI, coronary heart disease, hyperlipidemia, and diabetes. Q, quartiles; HEI-2015, Healthy Eating Index-2015; PD, Parkinson’s disease; BMI, body mass index; OR, odds ratio; CI, confidence intervals; Ref, reference.

In addition, a sensitivity analysis using unweighted logistic regression confirmed the stability of these results (Supplementary Table S4).

To validate the robustness of these findings, excluding outliers (HEI-2015 scores outside mean ± 3SD) did not change the results. In Model 4, each 10-point increase in HEI-2015 score was still associated with a lower PD risk (OR = 0.859, 95% CI: 0.743–0.993, *p* = 0.040). Participants in the highest HEI-2015 quartile (Q4) remained less likely to develop PD (OR = 0.559, 95% CI: 0.319–0.780, *p* = 0.042) (Supplementary Table S5).

These analyses confirm the robustness and consistency of the findings, highlighting the protective role of higher HEI-2015 scores against PD risk.

## 4 Discussion

The present study investigates the relationship between HEI-2015 and PD risk using a sizable, nationally representative sample from the NHANES database. Our findings demonstrate that a 14.2% decrease in PD prevalence was related to a 10-point rise in the HEI-2015 score, suggesting that higher diet quality may reduce PD risk. The prevalence of PD was 48.2% lower among participants in the top HEI-2015 quartile than those in the lowest quartile. These findings remained robust after adjusting for demographic, lifestyle, and health-related variables and were consistent across various sensitivity analyses.

Previous studies have examined the effects of specific dietary categories or nutrients on PD risk. However, our findings suggest that overall diet quality, as assessed by HEI-2015, is associated with a lower risk of PD. Notably, our study identified race and hyperlipidemia as potential effect modifiers in the correlation between HEI-2015 and PD, indicating that the protective effect of diet quality may vary by these factors.

Our findings align with a comprehensive review of prior literature, including 24 studies, that identified compounds like kaempferol in fruits and vegetables as having neuroprotective potential (36). Additionally, Satyam et al. (37) proposed that the intake of seafood and plant-based proteins helps support endogenous antioxidant systems, thereby protecting neuronal components from oxidative stress and reducing the risk of neurodegenerative diseases. Furthermore, evidence from recent studies suggests that *Vicia faba* L. (broad beans) demonstrates potential neuroprotective properties against PD (38, 39). A recent cross-sectional study using data from the NHANES database on American, reveals that excessive sugar consumption is positively associated with PD risk. This finding is consistent with the HEI-2015 guidelines, which recommend moderating sugar intake, and aligns with our research results (40). However, our study found no significant correlation between whole grains and PD risk, which contrasts with a large prospective study involving 131,368 individuals suggesting a protective role for whole grains (41). The observed discrepancy may result from differences in study design, the populations studied, or the methods used to assess dietary patterns, which requires further investigation.

The mechanisms underlying the protective role of diet quality in PD remain unclear. One proposed mechanism involves the regulation of oxidative stress and inflammation. Many of the bioactive compounds found in vegetables, fruits, fish, and beans—such as flavonoids—are known to modulate oxidative stress pathways, including NRF-2 and NADPH oxidase, elevate dopamine levels in the striatum, which are critical for reducing neuroinflammation (36). For example, quercetin, a flavonoid found in vegetables and fruits, has been shown to alleviate oxidative stress and protect against neurodegeneration in animal models of PD (42). Additionally, dietary patterns rich in antioxidants from vegetables and fruits, as well as seafood and lean proteins, may help protect or repair neuronal components from oxidative damage. This repair mechanism could prevent lipid, protein, and DNA damage in neurons, which are crucial to maintaining healthy brain function and preventing the onset of PD (37). Previous study reveals that excessive sodium intake exacerbates neuroinflammation and oxidative stress, contributing to the pathological processes underlying PD (43). However, as we found in our study, the relationship between sodium and PD may vary depending on the level of sodium intake, with moderate consumption potentially having different effects on PD risk.

Interestingly, our study found that race and hyperlipidemia may influence the relationship between HEI-2015 and PD. The association was stronger among individuals without hyperlipidemia, suggesting that metabolic health may influence the protective effect of diet. Individuals with hyperlipidemia may already be at an elevated risk for neurodegenerative diseases, and thus dietary interventions may not have the same protective effect. Additionally, differences in dietary patterns across racial groups could further modulate the effects of diet on PD risk. These findings highlight the significance of taking metabolic and demographic parameters into account in future research on diet and PD risk.

Our study has several notable strengths. One of the major strengths is the use of a large, nationally representative cohort from the NHANES database. This allows the findings to be generalized to the broader U.S. adult population, enhancing the external validity of our results. A variety of confounders are also taken into consideration in the research design, including age, sex, race, socioeconomic status, and comorbidities, which strengthens the validity of the observed associations. Additionally, the use of the HEI-2015, a validated and comprehensive dietary index, provides a more detailed and comprehensive understanding of overall dietary quality than traditional single-nutrient analyses. Finally, our study examined the relationship between PD and HEI-2015 scores, considering various levels of dietary quality and performing multiple sensitivity analyses to ensure the consistency of this association.

Despite its strengths, the research includes a few limitations. First, due to its cross-sectional design, causal relationships between diet quality and PD risk cannot be established. Second, consistent with previous research (6, 8, 9, 30, 31, 44), PD assessment in the NHANES dataset was based on medication use. While this approach offers a more objective measure compared to self-reported diagnoses, it may fail to identify individuals with PD who are not receiving treatment, potentially leading to the underestimation of PD prevalence. Future studies should integrate additional clinical diagnostic criteria to enhance classification accuracy. Third, while NHANES provides a high-quality and comprehensive dataset, the use of secondary data limits the ability to fully account for all potential confounders, introducing the possibility of unmeasured biases. Fourth, although NHANES is designed to be representative of the U.S. adult population, the findings may not be generalizable to other populations or geographic regions. Future studies in diverse populations and regions are needed to confirm the broader relevance of these findings. Lastly, there may be recall bias because the dietary consumption data came from two 24-h recalls. Given the current findings and limitations, further validation through large-scale cohort studies is necessary.

## 5 Conclusion

In conclusion, this study provides evidence that a higher-quality diet, as measured by the HEI-2015, is associated with a reduced risk of PD. Future studies are required to verify the relationship and investigate the mechanisms.

## Data Availability

The original contributions presented in the study are included in the article/Supplementary material, further inquiries can be directed to the corresponding author.

## Footnotes

^1^ https://www.cdc.gov/nchs/nhanes/

^2^ https://wwwn.cdc.gov/nchs/nhanes/analyticguidelines.aspx

^3^ https://www.cdc.gov/nchs/nhanes/

## References

[1] Tanner CM Ostrem JL. Parkinson’s disease. N Engl J Med. (2024) 391:442–52. doi: 10.1056/NEJMra2401857, PMID: 39083773

[2] Bloem BR Okun MS Klein C. Parkinson’s disease. Lancet. (2021) 397:2284–303. doi: 10.1016/S0140-6736(21)00218-X, PMID: 33848468

[3] Dorsey ER Elbaz A Nichols E Abbasi N Abd-Allah F Abdelalim A. Global, regional, and national burden of parkinson’s disease, 1990–2016: a systematic analysis for the global burden of disease study 2016. Lancet Neurol. (2018) 17:939–53. doi: 10.1016/S1474-4422(18)30295-3, PMID: 30287051 PMC6191528

[4] Yang W Hamilton JL Kopil C Beck JC Tanner CM Albin RL. Current and projected future economic burden of parkinson’s disease in the U.S. NPJ Parkinson’s Dis. (2020) 6:15. doi: 10.1038/s41531-020-0117-1, PMID: 32665974 PMC7347582

[5] Mittal P Dhankhar S Chauhan S Garg N Bhattacharya T Ali M. A review on natural antioxidants for their role in the treatment of Parkinson’s disease. Pharmaceuticals. (2023) 16:908. doi: 10.3390/ph16070908, PMID: 37513820 PMC10385773

[6] Cheng X Wu T Han L Sun T Huang G. Association between added sugars intake and parkinson’s disease status in U.S. adults: a cross-sectional study from NHANES 1990-2020. Arch Public Health. (2024) 82:225. doi: 10.1186/s13690-024-01445-8 39593073 PMC11590255

[7] Liu L Shen Q Bao Y Xu F Zhang D Huang H. Association between dietary intake and risk of parkinson’s disease: cross-sectional analysis of survey data from NHANES 2007-2016. Front Nutr. (2023) 10:1278128. doi: 10.3389/fnut.2023.1278128, PMID: 38192644 PMC10773772

[8] Zhang L Yang S Liu X Wang C Tan G Wang X. Association between dietary niacin intake and risk of parkinson’s disease in US adults: cross-sectional analysis of survey data from NHANES 2005-2018. Front Nutr. (2024) 11:1387802. doi: 10.3389/fnut.2024.1387802, PMID: 39091685 PMC11291445

[9] Su J Liu L Wang R Li C Wang Z Xu Q. Association between dietary β-carotene intake with parkinson’s disease and all-cause mortality among american adults aged 40 and older (NHANES 2001-2018). Front Nutr. (2024) 11:1430605. doi: 10.3389/fnut.2024.1430605, PMID: 39403400 PMC11472250

[10] Xu S Li W Di Q. Association of dietary patterns with parkinson’s disease: a cross-sectional study based on the United States national health and nutritional examination survey database. Eur Neurol. (2023) 86:63–72. doi: 10.1159/000527537, PMID: 36470220

[11] Tu X Wu N Wan Y Gan J Liu Z Song L. Association of dietary selenium intake and all-cause mortality of parkinson’s disease and its interaction with blood cadmium level: a retrospective cohort study. BMC Geriatr. (2024) 24:415. doi: 10.1186/s12877-024-05000-6, PMID: 38730347 PMC11088170

[12] Cen Y Wang L Zhang S Li X Xu Y Zeng Z. Correlations between dietary magnesium consumption and magnesium depletion score in relation to parkinson’s disease: a population-based study. Biol Trace Elem Res. (2024). doi: 10.1007/s12011-024-04428-6 39465480

[13] Zeng Z Cen Y Xiong L Hong G Luo Y Luo X. Dietary copper intake and risk of parkinson’s disease: a cross-sectional study. Biol Trace Elem Res. (2024) 202:955–64. doi: 10.1007/s12011-023-03750-9, PMID: 37462848 PMC10803382

[14] Hao X Li H Li Q Gao D Wang X Wu C. Dietary vitamin E intake and risk of parkinson’s disease: a cross-sectional study. Front Nutr. (2023) 10:1289238. doi: 10.3389/fnut.2023.1289238, PMID: 38249609 PMC10799344

[15] PMC. Prospective study of dietary pattern and risk of parkinson disease. (2024). Available online at: https://pmc.ncbi.nlm.nih.gov/articles/PMC2225168/ (Accessed December 7, 2024).

[16] Home. Dietary guidelines for americans. (2024). Available online at: https://www.dietaryguidelines.gov/ (Accessed December 2, 2024).

[17] Krebs-Smith SM Pannucci TE Subar AF Kirkpatrick SI Lerman JL Tooze JA. Update of the healthy eating index: HEI-2015. J Acad Nutr Diet. (2018) 118:1591–602. doi: 10.1016/j.jand.2018.05.021, PMID: 30146071 PMC6719291

[18] Zhou J Lou L Jin K Ye J. Association between healthy eating Index-2015 and age-related cataract in American adults: a cross-sectional study of NHANES 2005–2008. Nutrients. (2023) 15:98. doi: 10.3390/nu15010098, PMID: 36615757 PMC9823857

[19] Yin S Wang J Bai Y Yang Z Cui J Xiao Y. Association between healthy eating Index-2015 and kidney stones in American adults: a cross-sectional analysis of NHANES 2007–2018. Front Nutr. (2022) 9:820190. doi: 10.3389/fnut.2022.820190 35685877 PMC9172846

[20] Wang K Wu J Deng M Tao F Li Q Luo X. Associations of healthy eating index-2015 with osteoporosis and low bone mass density in postmenopausal women: a population-based study from NHANES 2007–2018. Front Nutr. (2024) 11:1388647. doi: 10.3389/fnut.2024.1388647, PMID: 38694220 PMC11061362

[21] Di XP Yuan C Wei X. Association between healthy eating Index-2015 and prostate enlargement: a cross-sectional study of the national and nutrition examination survey 2001-2008. Food. Nutr Res. (2024) 68:68. doi: 10.29219/fnr.v68.10828, PMID: 39239456 PMC11375444

[22] Hao X Liu G Li D. Association of healthy eating index-2015 and overactive bladder: a cross-sectional study. Front Nutr. (2024) 11:1400398. doi: 10.3389/fnut.2024.1400398 39355559 PMC11442424

[23] Ataei Kachouei A Kamrani F Haghighatdoost F Mohammadifard N Najafi F Farshidi H. Relationship of the prime diet quality score (PDQS) and healthy eating index (HEI-2015) with depression and anxiety: a cross-sectional study. BMC Public Health. (2024) 24:2919. doi: 10.1186/s12889-024-20369-0, PMID: 39438905 PMC11494750

[24] Li Y Qiu L Zhang C. Healthy dietary pattern improves cognitive function in elderly persons with periodontitis: A cross-sectional study of NHANES. Int Dent J. (2025) 75:545–53. doi: 10.1016/j.identj.2024.07.1207 39153892 PMC11976594

[25] Zoellner ER Patterson MA Sharrief AZ Savitz SI Tucker WJ Miketinas DC. Dietary intake and quality among stroke survivors: NHANES 1999-2018. J Nutr. (2023) 153:3032–40. doi: 10.1016/j.tjnut.2023.08.015, PMID: 37598751

[26] Cdc. About NHANES. National Health and Nutrition Examination Survey. (2024). Available at: https://www.cdc.gov/nchs/nhanes/about/index.html (Accessed 22 May 2025).

[27] Zhang Z Wang P Cui G Li H. Higher HEI-2015 score is associated with reduced risk of fecal incontinence: insights from a large cross-sectional study. BMC Public Health. (2024) 24:3221. doi: 10.1186/s12889-024-20729-w, PMID: 39567930 PMC11577588

[28] Yuguang L Chang Y Li H Li F Zou Q Liu X. Inflammation mediates the relationship between diet quality assessed by healthy eating index-2015 and metabolic syndrome. Front Endocrinol. (2024) 15:850. doi: 10.3389/fendo.2024.1293850, PMID: 38379861 PMC10877714

[29] Wu XF Yin F Wang GJ Lu Y Jin RF Jin DL. Healthy eating index-2015 and its association with the prevalence of stroke among US adults. Sci Rep. (2024) 14:3516. doi: 10.1038/s41598-024-54087-9, PMID: 38347074 PMC10861484

[30] Zeng Z Cen Y Wang L Luo X. Association between dietary inflammatory index and Parkinson’s disease from National Health and nutrition examination survey (2003–2018): a cross-sectional study. Front Neurosci. (2023) 17:1203979. doi: 10.3389/fnins.2023.1203979, PMID: 37547135 PMC10398569

[31] Ke L Zhao L Xing W Tang Q. Association between parkinson’s disease and cardiovascular disease mortality: a prospective population-based study from NHANES. Lipids Health Dis. (2024) 23:212. doi: 10.1186/s12944-024-02200-2, PMID: 38965560 PMC11223358

[32] Su J Liu L Wu D Wang R Wang Z Fan E. Association between serum total bilirubin with Parkinson’s disease among American adults (NHANES 1999 to 2018). Heliyon. (2024) 10:e36053. doi: 10.1016/j.heliyon.2024.e36053, PMID: 39224283 PMC11366891

[33] Chen L Cai M Li H Wang X Tian F Wu Y. Risk/benefit tradeoff of habitual physical activity and air pollution on chronic pulmonary obstructive disease: findings from a large prospective cohort study. BMC Med. (2022) 20:70. doi: 10.1186/s12916-022-02274-8, PMID: 35220974 PMC8883705

[34] Liang J Huang S Jiang N Kakaer A Chen Y Liu M. Association between joint physical activity and dietary quality and lower risk of depression symptoms in US adults: cross-sectional NHANES study. JMIR Public Health Surveill. (2023) 9:e45776. doi: 10.2196/45776, PMID: 37163324 PMC10209797

[35] Johnson CL Paulose-Ram R Ogden CL Carroll MD Kruszon-Moran D Dohrmann SM. National health and nutrition examination survey: Analytic guidelines, 1999–2010. Vital Health Stat. (2013) 161:1–24. 25090154

[36] Chaubey S Singh L. Deciphering the mechanisms underlying the neuroprotective potential of kaempferol: a comprehensive investigation. Naunyn-Schmiedeberg’s Arch Pharmacol. (2024) 398:2275. doi: 10.1007/s00210-024-03515-8 39414700

[37] Cannas C Lostia G Serra PA Peana AT Migheli R. Food and food waste antioxidants: Could they be a potent Defence against Parkinson’s disease? Antioxidants. (2024) 13:645. doi: 10.3390/antiox13060645 38929084 PMC11200518

[38] Abdel-Sattar E Mahrous EA Thabet MM Elnaggar DMY Youssef AM Elhawary R. Methanolic extracts of a selected egyptian vicia faba cultivar mitigate the oxidative/inflammatory burden and afford neuroprotection in a mouse model of parkinson’s disease. Inflammopharmacology. (2021) 29:221–35. doi: 10.1007/s10787-020-00768-6, PMID: 33118083

[39] Hu D Qing G Liu X Cheng J Zhang K He L. A study and in vitro evaluation of the bioactive compounds of broad bean sprouts for the treatment of parkinson’s syndrome. Molecules. (2024) 29:5160. doi: 10.3390/molecules29215160, PMID: 39519801 PMC11547941

[40] Wikipedia. Dietary guidelines for americans. (2025). Available online at: https://en.wikipedia.org/w/index.php?title=Dietary_Guidelines_for_Americans&oldid=1277941647 (Accessed March 31, 2025).

[41] Gao X Chen H Fung TT Logroscino G Schwarzschild MA Hu FB. Prospective study of dietary pattern and risk of parkinson disease. Am J Clin Nutr. (2007) 86:1486–94. doi: 10.1093/ajcn/86.5.1486, PMID: 17991663 PMC2225168

[42] de Oliveira VC Marinho MAG da Silva MM Hort MA Cordeiro MF Horn AP. Effects of quercetin in preclinical models of parkinson’s disease: a systematic review. Basic Clin Pharmacol Toxicol. (2024) 135:3–22. doi: 10.1111/bcpt.14011, PMID: 38682342

[43] Liu YZ Chen JK Li ZP Zhao T Ni M Li DJ. High-salt diet enhances hippocampal oxidative stress and cognitive impairment in mice. Neurobiol Learn Mem. (2014) 114:10–5. doi: 10.1016/j.nlm.2014.04.010, PMID: 24752150

[44] Huang W Xiao Y Zhang L Liu H. Association between a body shape index and parkinson’s disease: a large cross-sectional study from NHANES. Heliyon. (2024) 10:e26557. doi: 10.1016/j.heliyon.2024.e26557, PMID: 38420444 PMC10900994

