# Supplementary material for: Higher HEI-2015 score is associated with reduced risk of Parkinson’s disease: a nationwide population-based study
Source: Front Nutr. 2025 May 30;12:1541271. doi: 10.3389/fnut.2025.1541271 (PMC12162961; doi:10.3389/fnut.2025.1541271)
Supplement: Supplementary file 2 [file Table_2.DOC]

**Table S2.** Characteristics of participants in the NHANES 2003-2018 cycles, unweighted.

| **Characteristics** | **Total** | | | ***P*-value** |
| --- | --- | --- | --- | --- |
|  | **Overall**  **n=29581** | **Non-PD**  **n=29295** | **PD**  **n=286** |  |
| **Age** (years), mean (SD) | 50.34 (17.77) | 50.23 (17.75) | 61.21 (16.01) | <0.0001 |
| **Sex**, n (%) |  |  |  |  |
| Male | 14548.00 (49.18) | 14418.00 (49.22) | 130.00 (45.45) | 0.2067 |
| Female | 15033.00 (50.82) | 14877.00 (50.78) | 156.00 (54.55) |  |
|  |  |  |  |  |
| **Race,** n (%) |  |  |  |  |
| Non-Hispanic White | 13934.00 (47.10) | 13745.00 (46.92) | 189.00 (66.08) | <0.0001 |
| Non-Hispanic Black | 6185.00 (20.91) | 6143.00 (20.97) | 42.00 (14.69) |  |
| Mexican American | 4425.00 (14.96) | 4400.00 (15.02) | 25.00 ( 8.74) |  |
| Other Hispanic | 2399.00 ( 8.11) | 2379.00 ( 8.12) | 20.00 ( 6.99) |  |
| Other | 2638.00 ( 8.92) | 2628.00 ( 8.97) | 10.00 ( 3.50) |  |
| **Marital status**, n (%) |  |  |  |  |
| Married or Living  with a partner | 17911.00 (60.55) | 17750.00 (60.59) | 161.00 (56.29) | 0.1476 |
| Living alone | 11670.00 (39.45) | 11545.00 (39.41) | 125.00 (43.71) |  |
| **Family income**, n (%) |  |  |  |  |
| ≤1.30 | 8771.00 (29.65) | 8666.00 (29.58) | 105.00 (36.71) | 0.0032 |
| 1.31-3.50 | 11357.00 (38.39) | 11242.00 (38.38) | 115.00 (40.21) |  |
| >3.50 | 9453.00 (31.96) | 9387.00 (32.04) | 66.00 (23.08) |  |
| **Education level**, n (%) |  |  |  |  |
| Less than high school | 6699.00 (22.65) | 6621.00 (22.60) | 78.00 (27.27) | 0.1371 |
| High school or equivalent | 6910.00 (23.36) | 6843.00 (23.36) | 67.00 (23.43) |  |
| Above high school | 15972.00 (53.99) | 15831.00 (54.04) | 141.00 (49.30) |  |
| **Smoking status**, n (%) |  |  |  |  |
| Never | 15990.00 (54.05) | 15851.00 (54.11) | 139.00 (48.60) | 0.1951 |
| Former | 7626.00 (25.78) | 7545.00 (25.76) | 81.00 (28.32) |  |
| Now | 5965.00 (20.16) | 5899.00 (20.14) | 66.00 (23.08) |  |
| **Drinking status**, n (%) |  |  |  |  |
| Never | 3895.00 (13.17) | 3853.00 (13.15) | 42.00 (14.69) | <0.0001 |
| Former | 5768.00 (19.50) | 5674.00 (19.37) | 94.00 (32.87) |  |
| Mild | 9922.00 (33.54) | 9824.00 (33.53) | 98.00 (34.27) |  |
| Moderate | 4471.00 (15.11) | 4446.00 (15.18) | 25.00 ( 8.74) |  |
| Heavy | 5525.00 (18.68) | 5498.00 (18.77) | 27.00 ( 9.44) |  |
| **BMI**（kg/m^2^）,mean (SD) | 29.28 (6.94) | 29.27 (6.94) | 30.56 (7.20) | 0.0038 |
| **Coronary heart disease**,  n (%) |  |  |  |  |
| No | 28255.00 (95.52) | 27995.00 (95.56) | 260.00 (90.91) | 0.0003 |
| Yes | 1326.00 ( 4.48) | 1300.00 ( 4.44) | 26.00 ( 9.09) |  |
| **Hyperlipidemia**,n (%) |  |  |  |  |
| No | 8693.00 (29.39) | 8632.00 (29.47) | 61.00 (21.33) | 0.0055 |
| Yes | 20888.00 (70.61) | 20663.00 (70.53) | 225.00 (78.67) |  |
| **Diabetes**, n (%) |  |  |  |  |
| No | 24157.00 (81.66) | 23958.00 (81.78) | 199.00 (69.58) | <0.0001 |
| Yes | 5424.00 (18.34) | 5337.00 (18.22) | 87.00 (30.42) |  |
| **HEI-2015_total_score** ,  mean (SD) | 53.740 (13.308) | 53.764 (13.314) | 51.272 (12.444) | 0.0008 |
| **Quartile(Q) of HEI-2015** |  |  |  |  |
| Q1 | 7532.00 (25.46) | 7448.00 (25.42) | 84.00 (29.37) | 0.0134 |
| Q2 | 7395.00 (25.00) | 7308.00 (24.95) | 87.00 (30.42) |  |
| Q3 | 7343.00 (24.82) | 7280.00 (24.85) | 63.00 (22.03) |  |
| Q4 | 7311.00 (24.72) | 7259.00 (24.78) | 52.00 (18.18) |  |
| **PA(MET-min/wk)**,  median [IQR] | 720.00  [0.00, 2880.00] | 720.00  [0.00, 2920.00] | 180.00  [0.00, 1680.00] | <0.0001 |

Notes: Mean ± SD for continuous variables, P-value was by survey-weighted linear regression. % for categorical variables, the P-value was by survey-weighted Chi-square test. Q1 represents the unhealthiest diet quality, and Q4 represents the healthiest diet quality. P < 0.05 presents a significant difference.

***Abbreviation:*** Q, quartiles; HEI-2015, Healthy Eating Index-2015; PD, Parkinson’s disease; BMI, body mass index.
