# Supplementary material for: Higher HEI-2015 score is associated with reduced risk of Parkinson’s disease: a nationwide population-based study
Source: Front Nutr. 2025 May 30;12:1541271. doi: 10.3389/fnut.2025.1541271 (PMC12162961; doi:10.3389/fnut.2025.1541271)
Supplement: Supplementary file 3 [file Table_3.DOC]

**Table S3.** Associations of HEI-2015 components with PD, weighted.

| **Components** | **Model 1** | | **Model 2** | | **Model 3** | | **Model 4** | |
| --- | --- | --- | --- | --- | --- | --- | --- | --- |
|  | **OR (95%CI)** | ***P*-value** | **OR (95%CI)** | ***P*-value** | **OR(95%CI)** | ***P*-value** | **OR (95%CI)** | ***P*-value** |
| **Adequacy components** |  |  |  |  |  |  |  |  |
| Total vegetables | 0.830(0.718–0.960) | 0.012 | 0.799(0.694-0.920) | 0.002 | 0.811(0.705–0.934) | 0.004 | **0.812(0.706–0.933)** | **0.004** |
| Greens and beans | 0.859(0.778–0.950) | 0.003 | 0.872(0.792–0.960) | 0.006 | 0.884(0.803–0.973) | 0.123 | **0.884(0.803–0.973)** | **0.013** |
| Total fruits | 1.037(0.946–1.136) | 0.437 | 0.984(0.892–1.085) | 0.741 | 1.007(0.906–1.120) | 0.891 | 1.007(0.906–1.119) | 0.991 |
| Whole fruits | 1.024 (0.934–1.123) | 0.606 | 0.971(0.875–1.077) | 0.570 | 0.992(0.886–1.111) | 0.889 | 0.991(0.886–1.109) | 0.880 |
| Whole grains | 0.995(0.941–1.053) | 0.870 | 0.964(0.908–1.023) | 0.221 | 0.974(0.916–1.035) | 0.393 | 0.974(0.916–1.035) | 0.388 |
| Total dairy | 1.03 4(0.981–1.091) | 0.208 | 1.029(0.974–1.087) | 0.311 | 1.031(0.976–1.089) | 0.269 | 1.032(0.977–1.089) | 0.254 |
| Total protein foods | 0.790(0.680–0.918) | 0.002 | 0.811(0.695–0.946) | 0.008 | 0.809(0.694–0.942) | 0.007 | **0.811(0.697–0.942)** | **0.007** |
| Seafood and plant proteins | 0.903(0.839–0.971) | 0.006 | 0.908(0.845–0.976) | 0.009 | 0.922(0.858–0.990) | 0.026 | **0.922(0.859–0.990)** | **0.027** |
| Fatty acids | 0.943(0.877–1.015) | 0.117 | 0.947(0.880–1.020) | 0.148 | 0.953(0.884–1.028) | 0.208 | 0.952(0.884–1.026) | 0.199 |
| **Moderation components** |  |  |  |  |  |  |  |  |
| Sodium | 1.075 (1.013–1.142) | 0.017 | 1.066(1.005–1.130) | 0.033 | 1.071(1.007–1.139) | 0.031 | **1.071(1.006–1.114)** | **0.031** |
| Refined grains | 0.987 (0.931–1.046) | 0.650 | 0.964(0.909–1.022) | 0.215 | 0.970(0.913–1.030) | 0.313 | 0.970(0.913–1.030) | 0.317 |
| Saturated fats | 0.979(0.919–1.042) | 0.497 | 0.987(0.924–1.054) | 0.689 | 0.998(0.932–1.068) | 0.956 | 0.997(0.933–1.067) | 0.938 |
| Added sugars | 0.939(0.893–0.987) | 0.013 | 0.927(0.882–0.975) | 0.003 | 0.941(0.894–0.990) | 0.021 | **0.941(0.893-0.991)** | **0.022** |

Model 1：adjusted for none.

Model 2：adjusted for age, sex, race, marital status, family income, and educational level.

Model 3: adjusted for age, sex, race, marital status, family income, educational level, smoking status, drinking status, physical activity, and BMI.

Model 4: adjusted for age, sex, race,marital status,family income, educational level, smoking status, drinking status, physical activity, BMI, coronary heart disease, hyperlipidemia, and diabetes.

Whole grains, dairy, fatty acids, and all moderation components ranged from 0-10. All other components ranged from 0-5.

***Abbreviations：***BMI, body mass index; OR, odds ratio; CI, confidence interval; HEI-2015, Healthy Eating Index-2015; PD, Parkinson’s disease.
