# Supplementary material for: Higher HEI-2015 score is associated with reduced risk of Parkinson’s disease: a nationwide population-based study
Source: Front Nutr. 2025 May 30;12:1541271. doi: 10.3389/fnut.2025.1541271 (PMC12162961; doi:10.3389/fnut.2025.1541271)
Supplement: Supplementary file 4 [file Table_4.DOC]

**Table S4.** Association between HEI-2015 and PD, unweighted.

| **Variables** | **Unweighted participants**  **/total participants, No.** | | **Model 1** | | **Model 2** | | **Model 3** | | **Model 4** | |
| --- | --- | --- | --- | --- | --- | --- | --- | --- | --- | --- |
|  | **Without**  **PD** | **With**  **PD** | **OR (95%CI)** | ***P*-value** | **OR (95%CI)** | ***P*-value** | **OR(95%CI)** | ***P*-value** | **OR (95%CI)** | ***P*-value** |
| **HEI-2015**  **Per 10-points increase** | 29295  /29581 | 286  /29581 | 0.866(0.792~0.947) | 0.002 | 0.795 (0.723-0.874) | <0.001 | 0.82 (0.744-0.903) | <0.001 | 0.858 (0.743-0.902) | <0.001 |
| **Quartile(Q) of HEI-2015** |  |  |  |  |  |  |  |  |  |  |
| Q1(＜44.264） | 7448/7532 | 84/286 | 1(Ref) |  |  |  | 1(Ref) |  | 1(Ref) |  |
| Q2(44.265-53.207) | 7308/7395 | 87 /286 | 1.056(0.781-1.427) | 0.725 | 0.945(0.696-1.282) | 0.714 | 0.980 (0.721-1.331) | 0.896 | 0.979 (0.720-1.331) | 0.892 |
| Q3(53.207-62.857) | 7280/7343 | 63 /286 | 0.767(0.553-1.065) | 0.114 | 0.631(0.451-0.883) | 0.007 | 0.675 (0.481-0.948) | 0.023 | 0.672(0.479-0.943) | 0.022 |
| Q4（≥62.858） | 7259/7311 | 52/286 | 0.635(0.449-0.899) | 0.010 | 0.475(0.331-0.683) | <0.001 | 0.527 (0.365-0.762) | <0.001 | 0.525(0.363-0.759) | <0.001 |
| *P* for trend |  |  |  | 0.003 |  | <0.001 |  | <0.001 |  | <0.001 |

Model 1：adjusted for none.

Model 2：adjusted for age, sex, race, marital status, family income, and educational level.

Model 3：adjusted for age, sex, race, marital status, family income, educational level, smoking status, drinking status, physical activity, and BMI.

Model 4：adjusted for age, sex, race, marital status, family income, educational level, smoking status, drinking status, physical activity, BMI, coronary heart disease, hyperlipidemia, and diabetes.

***Abbreviations:*** Q, quartiles; BMI, body mass index; HEI-2015, Healthy Eating Index-2015; OR, odds ratio; CI, confidence intervals; Ref: reference; PD, Parkinson’s disease.
