# Supplementary material for: Higher HEI-2015 score is associated with reduced risk of Parkinson’s disease: a nationwide population-based study
Source: Front Nutr. 2025 May 30;12:1541271. doi: 10.3389/fnut.2025.1541271 (PMC12162961; doi:10.3389/fnut.2025.1541271)
Supplement: Supplementary file 5 [file Table_5.DOC]

**Table S5.** Association between HEI-2015 and PD, removing subjects with HEI-2015 score (mean ± 3SD), weighted.

| **Variables** | **Model 1** | | **Model 2** | | **Model 3** | | **Model 4** | |
| --- | --- | --- | --- | --- | --- | --- | --- | --- |
|  | **OR (95%CI)** | ***P*-value** | **OR (95%CI)** | ***P*-value** | **OR(95%CI)** | ***P*-value** | **OR (95%CI)** | ***P*-value** |
| **HEI-2015**  **Per 10-points increase** | 0.882(0.779–0.999) | 0.0478 | 0.816(0.713–0.934) | 0.004 | 0.858(0.743–0.992) | 0.038 | 0.859(0.743–0.993) | 0.040 |
| **Quartile(Q) of HEI-2015** |  |  |  |  |  |  |  |  |
| Q1(＜44.086） | 1(Ref) |  |  |  | 1(Ref) |  | 1(Ref) |  |
| Q2(44.087-53.052) | 1.112(0.688–1.795) | 0.6631 | 1.003(0.619–1.626) | 0.989 | 1.106(0.657–1.735) | 0.789 | 1.068(0.655–1.740) | 0.791 |
| Q3(53.052-62.702) | 1.123(0.646–1.952) | 0.6789 | 0.927(0.525–1.639) | 0.793 | 1.051(0.571–1.934) | 0.871 | 1.048(0.573–1.917) | 0.878 |
| Q4（≥62.702） | 0.602(0.358–1.102) | 0.0556 | 0.469(0.273–0.805) | 0.006 | 0.561(0.321–0.981) | 0.043 | 0.559(0.319–0.780) | 0.042 |
| *P* for trend |  | 0.102 |  | 0.011 |  | 0.096 |  | 0.090 |

Model 1:adjusted for none.

Model 2:adjusted for age, sex, race, marital status, family income, and educational level.

Model 3:adjusted for age, sex, race, marital status, family income, educational level, smoking status, drinking status, physical activity, and BMI.

Model 4:adjusted for age, sex, race, marital status,family income, educational level, smoking status, drinking status, physical activity, BMI, coronary heart disease, hyperlipidemia, and diabetes.

***Abbreviations:*** Q, quartiles; BMI, body mass index; HEI-2015, Healthy Eating Index-2015; OR, odds ratio; CI, confidence intervals; Ref: reference;PD, Parkinson’s disease.
